# New and Rapid Analytical Method Using HPLC-MS Detection for Acrylamide Determination in Black Ripe Olives

**DOI:** 10.3390/foods12214037

**Published:** 2023-11-06

**Authors:** Mercedes Brenes-Álvarez, Eva María Ramírez, Manuel Brenes, Pedro García-García, Eduardo Medina, Concepción Romero

**Affiliations:** Food Biotechnology Department, Instituto de la Grasa (IG, CSIC), Building 46, Ctra. Utrera km 1, 41013 Seville, Spain; mbrenes@ig.csic.es (M.B.-Á.); evamariaramirez@ig.csic.es (E.M.R.); brenes@ig.csic.es (M.B.); pedrog@ig.csic.es (P.G.-G.); emedina@ig.csic.es (E.M.)

**Keywords:** black olive, acrylamide, liquid extraction, HPLC-MS, validation

## Abstract

The presence of acrylamide, a known human carcinogen, in various heated foods raises significant concerns among consumers. Therefore, the development of a good analytical method is of paramount interest to the scientific community. Keeping this in view, a rapid, simple, reliable, and low-cost analytical method was developed and validated for acrylamide quantification in black ripe olives. The method consisted of the water extraction of the compounds from crushed olives with the addition of (^13^C_3_)acrylamide as an internal standard. The quantification was performed using high-pressure liquid chromatography and mass detection with positive electrospray ionization. The limits of detection and quantification were determined to be 4 and 11 µg/kg, respectively. The developed method exhibited excellent results in terms of accuracy (98.4–104.8%) and intra- and inter-day precision limits, both less than 20%. This new method was carried out by analyzing 15 samples of Spanish commercial black ripe olives, revealing a wide range of values, from 79 to 1068 µg/kg of fruit. The new protocol reduces the analysis time to just one hour per sample versus the minimum 24 h required by gas chromatography and mass detection, meaning that it could be a good option for the routine analysis of acrylamide in black ripe olives, and may be extendable to the analysis of this compound in other foods.

## 1. Introduction

Since the discovery of acrylamide (AA) in food in 2002 [1], both researchers and governmental agencies (Food and Drug Administration (FDA), European Food Safety Authority (EFSA)) have started to gather data on the presence of this potentially toxic compound in many heated foods. Early reports by the FDA identified black ripe olives as a product with a high level of acrylamide [2], a finding later corroborated by a series of EFSA reports released between 2009 and 2015 [3]. Notably, the levels of acrylamide in black ripe olives have shown considerable variation, ranging from 34 to 2103 µg/kg, likely attributable to factors such as the olive cultivar and processing practices [2,4,5,6,7,8].

While acrylamide and its main metabolite, glycidamide, have been identified as probable carcinogens, their genotoxic effects on humans are now under discussion, but a tolerable daily intake has been proposed as a health-based guidance value [9]. The FDA published guidelines in 2016 aimed at assisting the food industry in reducing acrylamide levels [10], and the European Commission established benchmark levels and mitigation measures for several highly consumed food products in 2017, but black olives were excluded [11]. Subsequently, the European Commission called upon member states to monitor the presence of acrylamide in specific foods, including olives, with the intention of establishing benchmark levels and mitigation strategies in the future [12].

Black ripe olives are harvested when they have a yellowish-green color with pinkish hues. They are stored for months in an acidified solution and the darkening stage currently involves an alkaline treatment followed by continuous aeration washes of the olive/liquid mixture for several days, during which acid is added to neutralize excess alkali in the fruits. Subsequently, the darkened fruits are immersed in an iron salt solution (ferrous gluconate or lactate) to fix the black color formed. Finally, the olives are packed with a preserving liquid, which consists of brine with a low concentration of ferrous salt [13]. Due to the product being packed with a neutral pH (6–8 units), sterilization is mandatory to ensure microbiological stability and eliminate any toxins potentially formed during processing. The sterilization is carried out at temperatures of 121–125 °C to comply with the sterilization requirements of 15 F_0_ recommended by the International Olive Council [14] and the California Department of Health [5]. The acrylamide content depends on the total heat treatment (time and temperature) applied during this step, although both linear and exponential relationships between acrylamide formation and the heating intensity have been reported [5,15,16,17]. The main mechanism of the formation of acrylamide in many foods involves the condensation of reducing sugars with the free amino acid asparagine via the Maillard reaction [18]. Alternative acrylamide formation pathways have also been suggested in lipid-rich food [19]. However, studies conducted on black olives have ruled out the direct involvement of asparagine in the formation of acrylamide in this product [4,15]. Other pathways for acrylamide formation in black olives have been proposed, involving peptides, peptides bound to polyphenols [20], glucosamine, and N-acetyl-glucosamine [21], but without confirmed evidence of their significance in acrylamide formation in olives. Consequently, the recommended mitigation measures for most heated foods are not suitable for processed black olives. Alternative measures have been explored, including the preservation time of the fruit before the darkening stage, the duration of the sterilization step, the addition of olive leaf extract, and the style of product presentation [4,7,22,23].

Nevertheless, both competent authorities and food business operators have been tasked with monitoring the presence of acrylamide in table olives [12]. This necessitates the development of an accurate, robust and labor-efficient analytical method for routine analysis. It currently entails the aqueous extraction of acrylamide from food, solid-phase extraction (SPE) cleanup, and analysis using either LC-MS or GC-MS [24,25,26,27]. In the case of black ripe olives, GC-MS has been employed after the derivatization of the sample [4,15]. Additionally, SPE alone or in combination with Carrez reagents (potassium hexacyanoferrate (Carrez I) and zinc sulfate (Carrez II)) have been used for the HPLC-MS/MS or HPLC-MS-QQQ analysis of this substance [6,7,8,16,22]. A potentiometric electronic tongue has also been employed to detect and quantify acrylamide in tables olives [17]. The objective of this work was to develop a rapid, simple, reliable, and cost-effective method for analyzing acrylamide in black ripe olives using HPLC-MS. In addition, a total of 15 commercial black ripe olive samples were analyzed to provide data for EFSA’s planned future benchmark levels for acrylamide in this product.

## 2. Materials and Methods

### 2.1. Olives

Nine samples of black ripe olives from the Hojiblanca cultivar were provided by Agrosevilla SCA company (La Roda de Andalucía, Sevilla, Spain) in order to define a new, faster, and simpler analytical method. In addition, 15 commercial samples were purchased in local markets, selected from different cultivars (Hojiblanca and Cacereña) and styles (whole, pitted, and sliced) in order to validate the proposed new method.

### 2.2. Moisture Analysis

The water content of the fruits was determined by weighing 10 g of the crushed pulp and then oven drying this at 105 °C to constant weight [28]. This was necessary to calculate the acrylamide content in the olive pulp.

### 2.3. Analysis of Acrylamide in Olive Pulp by HPLC-MS

The proposed new method consisted of extracting acrylamide with ultrapure water. Olives were pitted and then homogenized in a blender. A portion of 5 g was mixed with 10 mL of ultrapure water and spiked with 100 µL of the internal standard, (^13^C_3_)acrylamide (10 mg/L). The mixture was homogenized for 2 min with an Ultra Turrax homogenizer. Finally, the extract was filtered through a filter paper (Whatman nº1440–110). The samples were extracted in triplicate and each aliquot was injected into the HPLC.

Exceptionally, the aqueous supernatant (1 mL) was clarified via the addition of Carrez reagents I and II. The sample was vortexed for 30 s and centrifuged (9000× *g* for 10 min at 4 °C). One mL of the new supernatant was applied to an SPE cartridge (Discovery DSC-Mixer Cation Exchager (packed bed comprises both octyl (C8) and benzene sulphonic acid (SCX) bondings), 300 mg; Supelco, Bellefonte, PA, USA) that had previously been conditioned with methanol and water. The SPE eluate was collected into a test tube where 2 mL of ultrapure water was applied to the cartridge, and the eluate was collected into the same test tube. In addition, other samples were not treated with the Carrez reagents, but were purified with the SPE cartridge.

All extracts were filtered through a 0.22 μm pore size nylon filter, and an aliquot (20 μL) was injected into the liquid chromatograph. The quantification of the acrylamide compound was performed using a HPLC-MS system that consisted of a Waters 2695 Alliance with a pump, column heater, and autosampler modules, and the detection was carried out with a mass single quadrupole detector (QDa, Waters, Milford, CT, USA). The QDa mass detector was operated in the positive mode (ESI+), the capillary voltage was set to 0.8 kV, the cone voltage was set to 15 V, and nitrogen was used as a nebulizer gas, with the de-solvation temperature set to 600 °C. A Spherisorb ODS-2 (5 μm, 25 cm × 4.6 mm i.d., Waters Inc.) column was used and the separation was achieved using an elution gradient with an initial composition of 100% water acidified with formic acid (0.06%) and 0% methanol. After 20 min, the concentration of the latter solvent was increased to 100% for 2 min; at 22–32 min, the content of mobile phase B was 100%; at 32–34 min, the content of mobile phase B decreased linearly from 100% to 0%; at 34–44 min, the content of mobile phase B was 0%; the total analysis time for HPLC-MS employed was 44 min. The flow rate was 0.4 mL/min and the column was kept at 35 °C. The mass spectra of acrylamide displayed major signals at *m/z* 75 and (^13^C_3_)acrylamide at *m/z* 72. A calibration curve was built using 14 standard solutions with different concentrations of acrylamide (0, 4.9, 9.8, 20.4, 40.7, 81.4, 122.2, 203.6, 325.8, 407.2, 509.0, 1018.0, 1425.2, 2036.0 µg/kg). The standard solutions were prepared in a similar way to the samples to be analyzed: 5 g of non-sterilized crushed pulp was mixed with 10 mL of each standard solution and spiked with 100 µL of the internal standard, (^13^C_3_)acrylamide (10 mg/L). The analyses were carried out in triplicate.

A second HPLC equipment was used with some samples to confirm the results obtained using the new proposed method. On this occasion, the detection was carried out with a MS-MS detector. The equipment used was composed of an Agilent liquid chromatography system (1200 Series) consisting of a binary pump (G1312A), degasser (G1379B), and autosampler (G1329A), connected to a triple quadrupole API 4500 mass spectrometer (Applied Biosystems, Foster City, CA, USA) using an electrospray ionization interface in positive ionization mode (ESI+). Compounds were separated on a Zorbax Eclipse XDB-C18 (150 × 4.6 mm, 5 µm) column from Agilent. As eluent A, ultrapure water with formic acid (0.1%) was used. As eluent B, methanol was employed. The mobile phase was delivered at 0.5 mL/min using the following gradient: at 0–15 min, the content of mobile phase B was 0%; at 15–17 min, the content of mobile phase B was increased linearly from 0% to 100%; at 17–20 min, the content of mobile phase B was 100%; at 20–22 min, the content of mobile phase B was decreased from 100% to 0%; and at 22–27 min, the content of mobile phase B was 0%. Mass spectrometric acquisition was performed using multiple reaction monitoring (MRM). Nitrogen was used as the collision gas, curtain gas, and carrier gas. The ion spray voltage was set to 1.5 kV and the capillary temperature was set to 700 °C. The fragment ions in MRM mode were produced via the collision-activated dissociation of selected precursor ions in the collision cell of the triple quadrupole, and the selected products were analyzed with the second analyzer of the instrument. Acrylamide was identified using the *m/z* 72 → 55.1 transition and (^13^C_3_)acrylamide was identified using the *m/z* 75 → 58.1 transition.

### 2.4. Analysis of Acrylamide in Olive Pulp by GC-MS

Acrylamide was analyzed according to the method described by Casado and Montaño [4], which is the reference method for acrylamide analysis in table olives. Using this protocol, the analyte was detected as 2-bromopropenamide via GC-MS and (^13^C_3_)acrylamide was used as an internal standard. The quantification of acrylamide compounds was performed using a GC-MS system that consisted of an Agilent 8860 gas chromatographer with a pump, column heater, and autosampler modules, and the detection was carried out with an Agilent 5977B mass-selective detector operated in selected ion monitoring (SIM) mode with positive electron impact ionization. The GC column was a VF-WAXms capillary column (30 m × 0.25 mm i.d., 0.25 μm film thickness; Agilent); 2 µL of the extract (sample or standard solution) was injected into the gas chromatograph using the splitless injection method, and the carrier gas was helium at a column head pressure of 42 kPa. The injector temperature was 200 °C, and the temperature program used was as follows: isothermal for 1 min at 65 °C, then the temperature increased by 15 °C/min to 170 °C, by 5 °C/min to 200 °C, followed by 40 °C/min to 240 °C, and isothermal for 15 min. The GC-MS interface transfer line was held at 280 °C. Under these conditions, the retention time of the acrylamide and (^13^C_3_)acrylamide derivatives was 12.9 min. The ions monitored were *m/z* 70, 149, and 151 for 2-bromopropenamide, and *m/z* 110 and 154 for 2-bromo(^13^C_3_)propenamide. The acrylamide in the samples was quantified using the ion at *m/z* 151 for 2-bromopropenamide and the ion at *m/z* 154 for 2-bromo(^13^C_3_)propenamide. The other ions at *m/z* 70, 110, and 149 were considered only for confirmation purposes. A calibration curve was built using seven standard solutions with different concentrations of acrylamide (5, 10, 20, 40, 60, 100, 200 µg/L) and with each solution containing 100 µg/L (^13^C_3_)acrylamide. The standard solutions were prepared in a similar way to the samples to be analyzed.

### 2.5. Method Validation

To validate the new proposed method, parameters such as linearity, the limit of detection (LOD), the limit of quantification (LOQ), accuracy, and intra-day and inter-day precision were evaluated. The accuracy was calculated with three standard samples (56, 128 and 2003 µg/kg) on the basis of the given formula (mean concentration found/real concentration) × 100; the intra-day and inter-day precision were estimated by calculating the relative standard deviation (RDS) for six replicates of four different samples with low and high concentrations of acrylamide (82, 423, 682 and 1370 µg/kg).

### 2.6. Statistical Analysis

Statistical analyses were performed using Statistica 8.0 software (Statsoft, Tulsa, OK, USA). One-way analysis of variance, ANOVA (Duncan’s test), was employed to compare mean values with a significance level of 95%.

## 3. Results and Discussion

### 3.1. Method Optimization

Initially, the quantification protocol was optimized using a 500 µg/L acrylamide standard solution. Looking for the simplicity of the method, the use of high-pressure liquid chromatography and ultraviolet detections at 205 and 220 nm was tested according to the methodology proposed by Crawford and Wang [6]. However, the results were not satisfactory, so a mass detector was chosen, quantifying the molecular ion of *m/z* 72 for acrylamide and *m/z* 75 for the isotopic derivative ((^13^C_3_)acrylamide), which was used as an internal standard. The first mobile phase assayed was the one used by Crawford and Wang [6]; this was water and methanol:acetonitrile (1:1), both acidified with formic acid (0.1%). This mobile phase was compared with other options: (i) water and methanol, both acidified with formic acid (0.1%); and (ii) water acidified with 0.06% formic acid and methanol. An isocratic regime was used for the separation of compounds, but various combinations of mobile phases were also tested (100% acidified water; 95% acidified water and 5% organic solvent; 80% acidified water and 20% organic solvent). Also, different column temperatures (30–40 °C) and mobile-phase flow rates (0.4–1 mL/min) were assessed.

**Figure 1:**
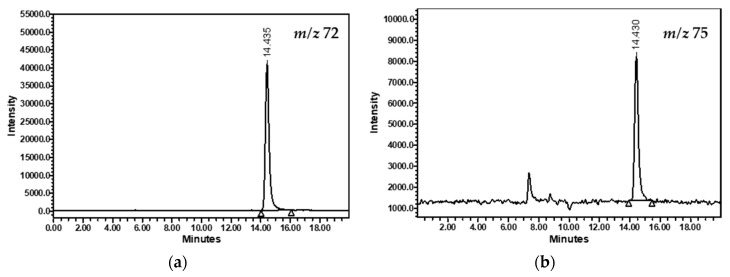
HPLC-MS chromatograms of acrylamide (**a**) and (^13^C_3_)acrylamide (**b**) from a sample of black ripe olives.

Figure 1 shows the chromatograms obtained with the final protocol selected: isocratic separation at 100% of acidified water with 0.06% formic acid at 35 °C, a flow rate of 0.4 mL/min and a classical ODS-2 analytical column. The compounds were clearly detected at 14.4 min.

**Figure 2:**
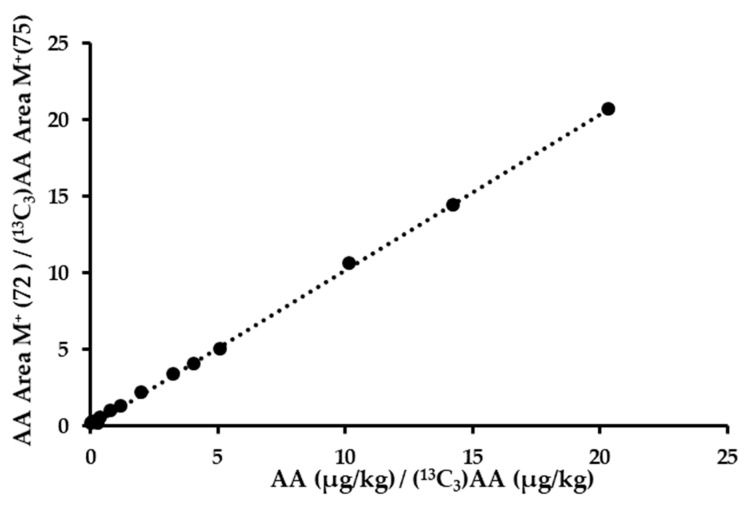
Calibration curve of acrylamide using the new proposed method.

A calibration line with 14 standard points (0–2036 µg/kg) was obtained (Figure 2); this represents the ratio of the areas obtained for acrylamide (*m/z* 72) and isotopic acrylamide (*m/z* 75) versus the ratio of the concentration of both molecules in µg/kg. A linear mathematical equation with a slope of 1.0186 and R^2^ of 0.9995 was obtained (Table 1).

The sample preparation protocol was also optimized, testing several alternatives. The first step was to define the extraction solvent, ultrapure water and ethyl acetate, obtaining better results with the former, which is a result that coincides with that reported by other authors [16,17]. In addition, other parameters were tested, including the ratio sample weight and solvent volume (1:1, 1:2, 1:5; g:mL), contact time of the preparation (2, 5, 10; minutes), homogenization method (ultraturrax, vortex, ultrasound), separation method (gravity, vacuum filtration, and centrifugation) and cleanup of the extract, similar to previous publications [7,8,22] (none, SPE, Carrez reagent + SPE). A derivatization step was not contemplated, although other researchers do propose it as an improvement for HPLC-MS quantification [25]. Overall, the final protocol was simplified to a single extraction with ultrapure water (1:2), homogenization with ultraturrax for 2 min, gravity filtration through filter paper and a second filtration of the aqueous phase through a nylon filter to remove possible traces of fats in the sample. This new protocol employs a short contact period as opposed to the 60 min proposed by other researchers [16,17], and it also avoids the use of one centrifugation step [16,17]. Prior to homogenization, the internal standard ((^13^C_3_)acrylamide) was added for subsequent quantification. It must be noted that the purification step was eliminated because a tendency to decrease the concentration of AA was observed in the samples when using both SPE and Carrez + SPE (Table 2). Moreover, it did not demonstrate any noticeable improvement for the chromatograms.

Once the method was defined, the results were confirmed with selective equipment HPLC-MS-MS equipment. Six samples of olives were prepared according to the new extraction protocol and quantified using both pieces of equipment, the routine machine (HPLC-MS) and the more selective (HPLC-MS/MS). The correlation between the results of both instruments was adjusted to the following mathematical equation Y_HPLC-MS_ = 1.1181X_HPLC-MS/MS_ − 0.045, with a value of R^2^ = 0.9905; this confirmed the good identification and quantification of acrylamide with the HPLC-MS methodology.

### 3.2. Validation of the New Method

Table 1 shows the validation parameters of this new method. The detection (LOD) and the quantification (LOQ) limits were evaluated according to Horwitz’s recommendations [29]. LOD and LOQ were considered to be 3 and 10 times, respectively, the standard deviation of the six blank samples analyzed. These values were calculated to be 4 and 11 µg/kg, respectively, which were limits lower than those reported by Casado and Montaño [4].

The accuracy and precision limits were evaluated according to the International Council for the Harmonization of Technical Requirements for Registration of Pharmaceuticals for Human Use (ICH) guideline [30]. The former parameter was calculated via the injection of three standard samples (56, 128 and 2003 µg/kg), the range values being 98.4–104.8%; this was more sensitive than the data previously reported in the available literature [4,26,31]. Moreover, this range of percentages is included in the criteria proposed in the ICH guideline [30], where an accepted limit of 80–120% is indicated.

The intra- and inter-day parameters were estimated by calculating the relative standard deviation for the analysis of six replicates of commercial black olives at four different concentrations (82, 423, 682 and 1370 µg/kg) on the same day (intra-) and six consecutive days (inter-). The intra-day repeatability was lower than the data showed by Rufián-Henares and Morales [31]. Both precision ranges were less than 20%, considering the upper limit value of this measure [30].

The analytical GREENness metric tool (AGREE) is a reliable greenness assessment method for determining the greenness of analytical methods in both qualitative and quantitative terms. The proposed new protocol yielded an AGREE score of 0.59 points on a scale from redness (0 points) to greenness (1 point), with Principles 4, 6, 10 and 12 being the highest rated in the protocol [32].

### 3.3. Comparison between the Proposed Method (HPLC-MS) and the Validated GC-MS

Until now, the reference method for acrylamide analysis in table olives has been that proposed by Casado and Montaño [4]. This method supposes numerous sample preparation steps including a water extraction, a cleanup via SPE cartridge, the formation of a brominated acrylamide derivative overnight at 4 °C, an extraction by ethyl acetate, solvent evaporation, and the solubilization of the analyte in ethyl acetate along with trimethylamine to form 2-bromopropenamide. The detection is carried out by using GC-MS. In contrast, the proposed method consisted of a single extraction with water and a double filtration of the aqueous solution, followed by detection with HPLC-MS. Hence, the analysis of acrylamide in black ripe olives took 24 h using the old GC-MS method, while it only took one hour using the proposed HPLC-MS method.

**Figure 3:**
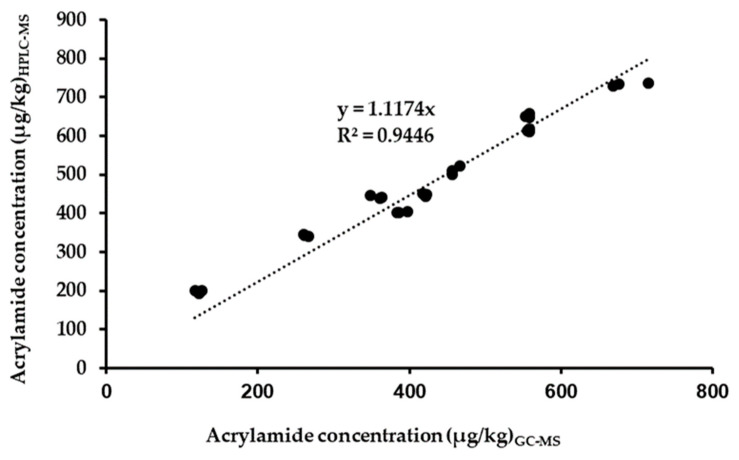
Correlation between acrylamide concentration (µg/kg) determined using gas chromatography with mass detection (GC-MS) vs. the liquid chromatography with mass detection (HPLC-MS) method.

Nine samples of black ripe olives were analyzed using both methodologies and the results were compared. Figure 3 shows that there is a good correlation, with an R^2^ value of 0.9446. The equation obtained shows that the new method has an overexpression of 12% with respect to the reference method. Therefore, the new method can be proposed for the routine analysis of acrylamide in table olives due to its simplicity and low cost; therefore, a high number of samples can be analyzed on a daily basis.

### 3.4. Analysis of Commercial Samples

Table 3 shows the results obtained using the proposed new analytical method on 15 samples of black ripe olives. The sampling was representative of the Spanish commercial product, analyzing the two most representative cultivars, Hojiblanca and Cacereña, and the three types of presentation: sliced, pitted, and whole. A high variability was observed within the results, and no trend either according to cultivar or the type of presentation was observed. The concentration ranged from 79 to 1068 µg/kg of fruit, which is in good agreement with previous studies that showed the AA in black ripe olives to be between 34 and 2103 µg/kg [2,4,5,6,7,8,33]. The mean value was in the order of 450 µg/kg of fruit with a standard deviation of 271 µg/kg of fruit. It should be noted that 40% of the results were included in the range of 300–600 µg/kg of fruit, which is the limit value recommended for other foods such as breakfast cereals, cookies, and roasted coffee [11] and according to the data reported in [34]. In general, the dispersion of the results with respect to the mean was not very high; in most samples, the value of the relative standard deviation was less than 10%.

The statistical analysis of these data (Duncan’s test) indicated no statistical differences among the olive style, and the higher content of acrylamide in olives of the Hojiblanca cultivar than Cacereña was in agreement with previous studies [7].

## 4. Conclusions

Currently, the analysis of acrylamide in black ripe olives is a laborious and expensive methodology. The search for a new, simpler, and more economical methodology has always been a challenge for researchers. A new method for acrylamide analysis in black olives has been developed and validated. It is simple, fast, and efficient, and consists of an easy extraction with water and a quantification via HPLC-MS, with the total protocol taking only one hour per sample. It must be taken into consideration that the cost of the HPLC-MS equipment is must lower than the HPLC-MS-MS equipment, potentially enabling the routine analysis of this substance by many laboratories all over the world. The application of the method to 15 samples of Spanish commercial olives showed a wide range in the concentration of acrylamide, from 79 to 1068 µg/kg of olive flesh. The proposed method should be considered by the analysis laboratories of both official public research organizations and private companies. The economy of time and money and the sustainability of the technology are common goals for all. In addition, this new method will allow us to advance more rapidly in our future research on the elucidation of the mechanism of acrylamide formation in table olives, which still remains unknown.

## Figures and Tables

**Table 1:** Validation parameters of the method.

Statistical	Value
Slope	1.0186
Coefficient of determination (R^2^)	0.9995
LOD (µg/kg)	4
LOQ (µg/kg)	11
Accuracy ^1^ (%)	98.4–104.8
Intraday precision ^2^ (RSD, %)	1.7–2.6
Interday precision ^2^ (RSD, %)	2.4–18.9

^1^ Determined via the analysis of three different standard solutions. ^2^ Determined via the analysis of four different samples with low and high concentrations of acrylamide.

**Table 2:** Effect of different precleaning approaches for the analysis of acrylamide in olives using the new proposed method. A: new extraction method proposed in 2.3 Materials and Methods section.

Sample	Alternatives	Acrylamide Concentration (µg/kg)	
		Value ^1^	Standard Deviation
Pitted olive	A	342 a ^2^	5
	A + SPE	323 a	12
	A + Carrez + SPE	327 a	19
Whole olive	A	540 a	17
	A + SPE	529 a	31
	A + Carrez + SPE	491 b	7

^1^ Values are the mean of triplicate analyses. ^2^ For each sample, column values followed by the same letter do not differ at the 5% level of significance according to Duncan’s multiple-range test.

**Table 3:** Content of acrylamide in Spanish black ripe olives. Data are from 15 commercial samples from different styles and cultivars.

Style	Cultivar	Acrylamide Concentration (µg/kg)		
		Value ^1^	Standard Deviation	Relative Standard Deviation
Sliced	Hojiblanca	720	39	5
		596	54	9
		515	14	3
		364	26	7
		263	46	17
Pitted	Hojiblanca	1068	174	16
		466	53	11
		326	14	4
	Cacereña	108	6	5
		79	5	6
Whole	Hojiblanca	765	39	5
		589	33	6
		450	15	3
	Cacereña	300	51	17
		148	13	9

^1^ Values are the mean of triplicate analyses.

## Data Availability

The data are contained within the article.
